# Viral load, not food availability or temperature, predicts colony longevity in an invasive eusocial wasp with plastic life history

**DOI:** 10.1038/s41598-021-89607-4

**Published:** 2021-05-12

**Authors:** Kevin J. Loope, Erin E. Wilson Rankin

**Affiliations:** 1 Department of Entomology, University of California, Riverside,, 900 University Ave., Riverside, CA 92507 USA; 2 Present Address: Department of Biology, Georgia Southern University, 4324 Old Register Road, Statesboro, GA 30460 USA

**Keywords:** Behavioural ecology, Invasive species

## Abstract

Social insect colonies exhibit a variety of life history strategies, from the annual, semelparous colonies of temperate bees and wasps to the long-lived colonies of many ants and honeybees. Species introduced to novel habitats may exhibit plasticity in life history strategies as a result of the introduction, but the factors governing these changes often remain obscure. *Vespula pensylvanica*, a yellowjacket wasp, exhibits such plasticity in colony longevity. Multi-year (perennial) colonies are relatively common in introduced populations in Hawaii, while source populations in the western United States are typically on an annual cycle. Here, we use experiments and observational data to examine how diet, disease, nest thermal environment, and nest location influence colony longevity in a population with both annual and perennial colonies. Counter to our predictions, experimental feeding and warming did not increase colony survival in the winter in the introduced range. However, Moku Virus load and wasp colony density predicted colony survival in one year, suggesting a potential role for disease in modulating colony phenology. We also found that local *V. pensylvanica* colony density was positively correlated with Moku Virus loads, and that *Arsenophonus* sp. bacterial loads in *V. pensylvanica* colonies were positively associated with proximity to feral honeybee (*Apis mellifera*) hives, suggesting potential transmission routes for these poorly understood symbionts. The factors influencing colony longevity in this population are likely multiple and interactive. More important than food availability, we propose winter precipitation as a critical factor that may explain temporal and spatial variation in colony longevity in these invasive wasps.

## Introduction

Natural selection frequently operates via survival and reproduction at the colony level in highly eusocial insects^1^. Thus, colonies themselves may possess evolved life history traits, in addition to the life history traits of individuals^2^. Colonies of the eusocial Hymenoptera exhibit a range of life history strategies, from the annual cycles of most temperate social bees and wasps to the perennial and long-lived colonies of ants, honeybees and many tropical wasps and bees^3^. Many factors influence social insect life history strategies: colony longevity may evolve in response to seasonal variation in temperature or resource availability, as well as pressure from predators, pathogens and resource competitors^2,4,5^.

The annual cycles of temperate species, such as vespine and polistine wasps, are a likely result of winters that constrain colony survival when foraging conditions and resources are limited, favoring a single colony reproductive event (semelparity) prior to overwintering by new daughter queens^2,5^. The transition from semelparity to iteroparity (multiple reproductive events) is thought to be quite difficult, given the costs of trading current for future reproduction and the risks of not surviving until a second reproductive event (Cole’s Paradox^4^). Despite a long evolutionary history of annual cycling, several populations of *Vespula* yellowjacket wasps exhibit remarkable variation in colony longevity. In such populations, some colonies persist into a second or third year and attain sizes that are orders of magnitude larger than their annual counterparts^6^—a dramatic departure from the recent ancestral state of strict annual cycling. This extension of colony life appears to be facilitated by the adoption of new queens (secondary polygyny), rather than the extension of the foundress queen’s lifespan^7,8^. Unsurprisingly, this occurs only in regions with warm winters, such as the southeastern USA and southern California, where *Vespula* spp. are native^9,10^, as well as in subtropical and Mediterranean climates where various *Vespula* spp. have been introduced, such as Hawaii, Australia and New Zealand^7,11,12^. Yet within these populations, the majority of colonies remain on an annual cycle. What are the proximate factors that drive variation in colony lifespan in such populations?

In this study, we test how resource availability, nest thermal environment, disease and colony spatial arrangement influence colony longevity in a population of the western yellowjacket, *Vespula pensylvanica,* introduced to the Big Island of Hawaii from the native range in the western United States. *Vespula pensylvanica* was first recorded on the Hawaiian Islands in 1919 and became widespread in the late 1970s, attaining high densities and causing widespread ecological damage^7,13^. Due to relatively benign winters, this population exhibits large variation in colony longevity, with up to 20% of colonies thought to be overwintered in some years, though the majority of colonies appear to remain on an annual cycle^6,7^. The expansion of the typical active season, and the outsized impact of giant perennial colonies^6^, mean that the phenotypic plasticity of invasive *V. pensylvanica* colonies has a direct impact on the ecological damage this species causes. Determining the factors that govern colony senescence could help to better understand and mitigate the damage caused by these notorious invaders. Longitudinal observations of colonies through the winter, even if they senesce prior to the subsequent growing season, may illuminate the processes that influence true perenniality; it seems likely that survival is contingent upon many interacting variables, and colonies that persist longer into the winter are more likely to survive through to the more favorable conditions in the following spring and summer. In other words, the factors that promote longer-lived annual colonies likely overlap with those that promote perenniality. Thus, data on what factors influence colony longevity, even for senescing annual colonies, could help us to better understand the processes leading to perenniality, given its relative rarity.

To explain variation in colony longevity, we first hypothesized that some *V. pensylvanica* colonies may exploit the abundant feral honeybee (*Apis mellifera*) hives that co-occur on the landscape (Wilson Rankin 2014). *Vespula pensylvanica* frequently scavenges and preys upon adult honeybees for protein^14^, and also robs hives for honey stores (K.J.L. and E.W.R, pers. obs.). Given that perennial honeybee hives present a constant and abundant food source throughout the year, we hypothesized that access to this resource may promote greater longevity of *V. pensylvanica* colonies located nearby^15^. To test this idea, we predicted an association between proximity to honeybee hives and colony longevity, and that colonies experimentally supplemented with honeybee protein and honey would exhibit increased longevity.

Second, we hypothesized that low temperatures in winter may accelerate colony die-off. To test for this, we experimentally raised ground surface temperatures for some colonies using passive solar heating with open plastic cones. We predicted that warmed colonies would live longer than colonies without experimental warming.

Third, we hypothesized that pathogens may limit *V. pensylvanica* colony survival, favoring a semelparous life history^4,5^. Numerous studies show that pathogens can have negative effects on social insects at the colony level (e.g., Refs^16–18^). Much recent work has focused on the role of social behaviors in mitigating the increased threat of pathogens in highly social insects^19^ and the role of agriculturally important eusocial insects (honeybees and bumblebees) in transmitting pathogens to wild populations^20–23^. However, studies linking pathogens to colony survival and reproduction in wild populations and in non-model species are rare^24^, and the degree to which pathogens influence wild populations is poorly understood. Although invasive species may experience relaxed pathogen pressure as a result of enemy release in a novel environment^25^, we know that numerous putative pathogens are present in invasive *Vespula*^26^, including *V. pensylvanica* wasps in Hawaii^27–29^. High pathogen loads could limit colony survival late in the season, particularly in species with an evolutionary history of an annual cycle. Innate and behavioral immune systems in annual-cycle species may evolve to permit the buildup of pathogens late in the season after new gynes and males have been produced, given that the window for further reproduction is closing as a result of the oncoming winter^30^. We predicted that high colony-level pathogen loads late in the season would be associated with decreased colony longevity going into the winter. We examined three possible pathogens. First, we quantified the colony-level load of the recently discovered Moku Virus (an *Iflavirus*) because it was first described in *V. pensylvanica* on the Big Island, exhibits high copy number in wasps, and is related to pathogenic viruses^27^. We also quantified *Arsenophonus* sp. load, a member of a genus of intracellular endosymbiotic bacteria common in insects that has been associated with poor health of honeybee hives^31^. Thirdly, we screened for trypanosomatids, which are common gut parasites of insects, including eusocial Hymenoptera (e.g., Schmid-Hempel and Tognazzo 2010).

Finally, we hypothesized that the proximity of wasp colonies to one another could influence colony survival, as well as colony pathogen load. Eusocial Hymenoptera are central place foragers, with workers’ foraging range constrained by the location of the nest to which they must return. *Vespula pensylvanica* workers typically remain close to the nest, with the majority of foraging occurring within a few hundred meters^33^. Thus, an effect on survival of close proximity to other wasp colonies may indicate that intraspecific competition for resources limit colonies when they become large, late in the season. Furthermore, we hypothesized that proximity to other wasp colonies, or possibly to honeybee hives, could increase exposure to horizontally transmitted pathogens. Social insect pathogens may be transmitted between conspecific colonies during drifting or raiding/robbing, as well as between non-nestmate workers on shared floral resources^34–36^. Similarly interspecific transmission is also possible^37^, and occurs at our study site between *V. pensylvanica* and honeybees^28^. We thus tested for effects of proximity to both wasp and honeybee colonies on colony-level pathogen loads.

## Methods

### Field site and nest discovery

**Figure 1:**
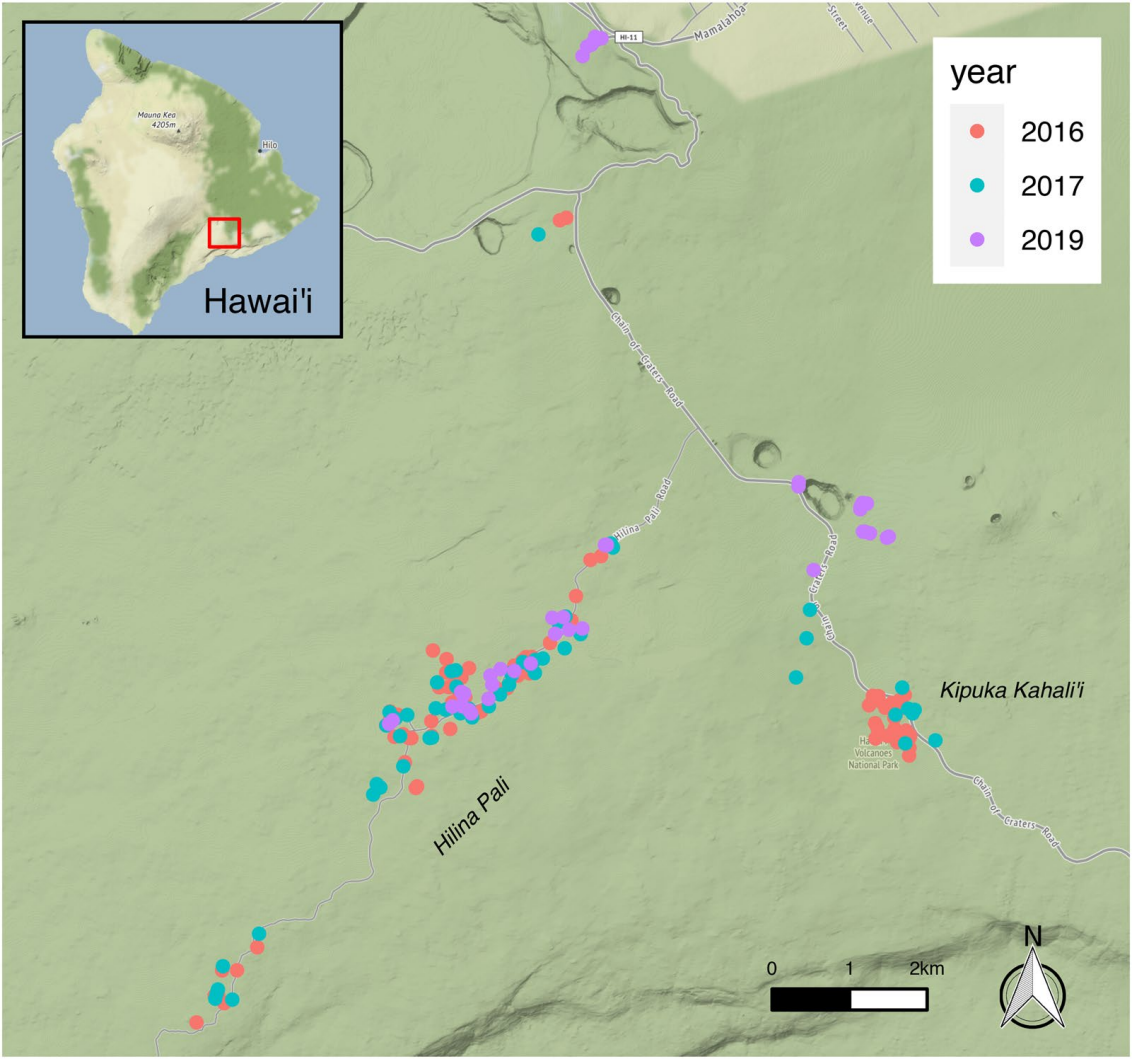
Locations of all colonies found in 2016, 2017 and 2019 in Hawaii Volcanoes National Park, on the Big Island of Hawaii. Most of the colonies in the study were located along Hilina Pali Road and at Kīpuka Kahali'i, in open 'Ohi'a forest approximately 850–1000 m above sea level. Basemap: Stamen Terrain (obtained through package *ggmap *^65^).

In September 2016, 2017 and 2019, we found *V. pensylvanica* and *A. mellifera* colonies at several sites in Hawaii Volcanoes National Park, Hawaii, USA. Fieldwork was not conducted in 2018 due to volcanic activity and resulting park closure. Our primary sites were Hilina Pali Rd (HP), and Kīpuka Kahali’i (KK), though in 2019 numerous colonies were found in other areas due to a lack of colonies at KK in that year (Fig. 1), possibly the result of volcanic gases released at nearby Pu'u o'o. Both HP and KK possess sparse 'Ohi'a lehua (*Metrosideros polymorpha*) forest mixed with open areas of volcanic rock, and lie 850-1000 m above sea level on the southeastern slope of Mauna Loa, ~ 8 km south of the Kilauea crater. In addition to the dominant 'Ohi'a, both the native Pūkiawe shrub (*Leptecophylla tameiameiae*) and the invasive faya bush (*Morella faya*) are also common. While the Hilina Pali site contains patches of older 'Ohi'a and grassy areas, recent volcanic activity at KK has resulted in exclusively young 'Ohi'a trees and a carpet of pea-sized volcanic gravel. Because of this, KK also lacks suitable honeybee nest cavities (see below), though dense forest along the northern edge may harbor hives. The sites receive 1300–2000 mm of rainfall per year^58^, with a cool and rainy season extending from November to March. Average daily highs and lows range from 23 °C and 13 °C in August to 20 °C and 9 °C in January.

*V. pensylvanica* colonies were discovered by placing canned chicken bait cups at regular intervals and following the attracted foragers back to the nest. Some honeybee hives were known from fieldwork at the site in 2015, and more were found during *V. pensylvanica* nest searching. In 2016 we systematically located hives using visual searches. To do so, we walked circular transects of 25 m, 75 m, 125 m and 175 m radius from each wasp colony without a known honeybee colony within 200 m, thus establishing estimates of the proximity of wasp colonies to honeybee colonies. The location of each nest was recorded using a Garmin GPSmap 64 s. All fieldwork and collections were conducted under permits HAVO-2016-SCI-0050 and HAVO-2019-SCI-0021.

### Diet manipulation

In mid-September 2016, we manipulated the availability of natural honeybee forage by removing wild honeybee colonies (n = 8) from the vicinity (200 m) of a subset of *V. pensylvanica* colonies at the HP site. This was done to increase the number of *V. pensylvanica* colonies distant from honeybee hives, as nearly all *V. pensylvanica* colonies at HP were originally within 200 m of a honeybee hive. These cavities were checked every few weeks to confirm that no new swarms re-occupied them. No colonies were removed in 2017 or 2019, given the lack of an effect of honeybee hive proximity in 2016 (see “Results” section).

Beginning in mid-September in all three years, we supplemented the diet of a subset of *V. pensylvanica* colonies with honeybee adults and honey, both collected from hives within or adjacent to the park boundary. Each week, fed colonies received 50 cm^3^ of frozen adult honeybees (~ 140 individuals), and 15 ml honey feeders were filled. This amount corresponds to the upper range of daily observed rate of foragers returning with naturally captured honeybee parts in a prior study of yellowjacket diet (~ 20 individuals/day^15^). For details of feeding methods, see Supplementary Methods.

### Manipulation of nest thermal environment

Beginning on approximately September 15 in 2017 and 2019, we placed passive, open-top solar warming cones (hereafter “cones”; diameter: 50 cm (top), 84.6 cm (bottom); Supplementary Fig. S6 and Supplementary Methods;^59^) around nest entrances. We used a crossed design in both years, with 10 cones placed at fed colonies and 10 cones placed at unfed colonies each year. In 2017, to verify that cones indeed warmed the nest entrance, we placed iButton temperature data loggers (Thermochron DS1921) approximately 5 cm into the nest entrance tunnel. iButtons recorded temperature every three hours for the duration of the field season. Nest entrances with cones experienced a 1.5 to 2.9 °C average warming effect for October through February, compared to controls (Supplementary Fig. S7). A similar study in California found that cones increased the maximum temperature of surface of *V. pensylvanica* nest surfaces by ~ 1.5 °C^15^, suggesting that this method warms the nest itself.

### Sample collection and pathogen quantification

On Sept 16–24, 2016 and Sept 25–27, 2017, we collected adult wasps from entrances of 76 colonies (2016) and 41 colonies (2017). Samples were collected into either ethanol or a liquid nitrogen-chilled dry shipper (2017), frozen and shipped to the lab, and then stored at − 80 ºC until processing. We used RT-qPCR to quantify relative load for Moku Virus^42^, qPCR to quantify relative load for *Arsenophonus* sp.^48^, and scored colonies for presence/absence of trypanosomatids^32^ using standard PCR and gel electrophoresis. We also scored a subset of colonies for Moku Virus replication by detection of the negative strand of viral RNA in our RNA extracts using standard methods^60^. For additional details, see Supplementary Methods.

### Survival monitoring and analysis

We monitored the foraging activity and survival of colonies every 1–3 weeks until a colony had two checks in a row with no forager traffic within a 4-min period in good weather. Colony traffic is a reliable indicator of colony size^38^, and sporadic subsequent checks on a subset of “zero-traffic” colonies verified this as a reliable indicator of colony death. Actual colony death date was estimated using the Mayfield-40% method^61^ as the date 40% of the duration between the last observation of the colony alive and the subsequent observation, and longevity was coded as the number of days survived past September 1.

All statistical analyses were performed in R v.4.0.2^62^. We used Cox survival models (function cox.ph) in the R package *survival*^63^ to analyze colony longevity. To test for an effect of experimental feeding, we ran a model containing all 3 years of data (n = 134 colonies; n = 53 for 2016, n = 41 for 2017; n = 40 for 2019), and including treatment (fed or not fed) and year as predictors. We then modeled each year’s survival separately, because the initial model suggested significant inter-annual differences and to permit testing for effects of additional variables for which we did not have data from every year (Table 1).

**Table 1:** Summary of analyses across the three years of study.

Response	Analysis of:	Method	2016	2017	2019
*Vespula* colony longevity	Experimental feeding	Cox regression	Y	Y	Y
	Proximity to honeybee hives	Cox regression	Y		
	Experimental heating	Cox regression		Y	Y
	Wasp pathogen loads	Cox regression	Y	Y	
	Wasp colony density	Cox regression	Y	Y	
*Vespula* colony pathogen load	Spatial autocorrelation	Moran’s I	Y	Y	
	Wasp colony density	GLM	Y	Y	
	Honeybee hive proximity	GLM	Y		

For data from 2016, we modelled survival for experimental colonies only (n = 53), and for a larger set that also included unmanipulated colonies (n = 74, excluding two colonies at the CRT site). Predictors were site (Kīpuka Kahali’i or Hilina Pali), feeding treatment (feed or control), trypanosomatids (presence or absence), *Arsenophonus* sp. (continuous relative load (on a log scale)), Moku load (high or low, threshold = relative load of 7 due to bimodality of load; see “Results” and Fig. 1), and the number of conspecific colonies within 100 m. To test for an effect of proximity to the nearest honeybee hive, we re-ran the model with all colonies with honeybee proximity coded as none (site KK), low (site HP, no hive within 200 m), or high (site HP, hive within 200 m). Again, we removed site as a predictor, because site is confounded with honeybee presence. For data from 2017, we used a single model with 37 experimental colonies and the same predictors as 2016, except that we removed trypanosomatids because none were detected in 2017 (four monitored colonies without pathogen data were excluded). For 2019 colonies, we used a single model with 40 colonies and treatment as a predictor. We verified that all models met the proportional hazards assumption using the function cox.zph().

### Colony spatial arrangement and pathogen load

We analyzed spatial effects on pathogen load for 2016 and 2017 colonies at HP and KK sites (Table 1; Fig. 1). For 2016 and 2017 data, we looked for spatial associations of Moku and *Arsenophonus* sp. loads using global Moran’s I tests with a k-nearest-neighbors definition of proximity, using Monte Carlo permutation tests (function moran.mc) in the package *spdep*^64^ with k = 1–4 neighbors. We tested for effects of conspecific nest density (the number of colonies within 100 m) on pathogen load using linear (*Arsenophonus* sp. loads) and binomial (high vs low Moku Virus loads) models with the function glm() (R Core Team 2020). Due to our methodical honeybee hive searches in 2016, we also checked for an effect of proximity to honeybee hives for 2016 only, comparing pathogen loads between colonies with no nearby honeybee hives, low honeybee availability, or high honeybee availability using linear (*Arsenophonus* sp.) and binomial (Moku Virus) models with the function glm(). We excluded four colonies at HP that lacked data on nearby honeybee hives. We excluded trypanosomatid presence from spatial analysis because only six colonies were positive in 2016, and zero were positive in 2017.

## Results

### Pathogen presence and load

**Figure 2:**
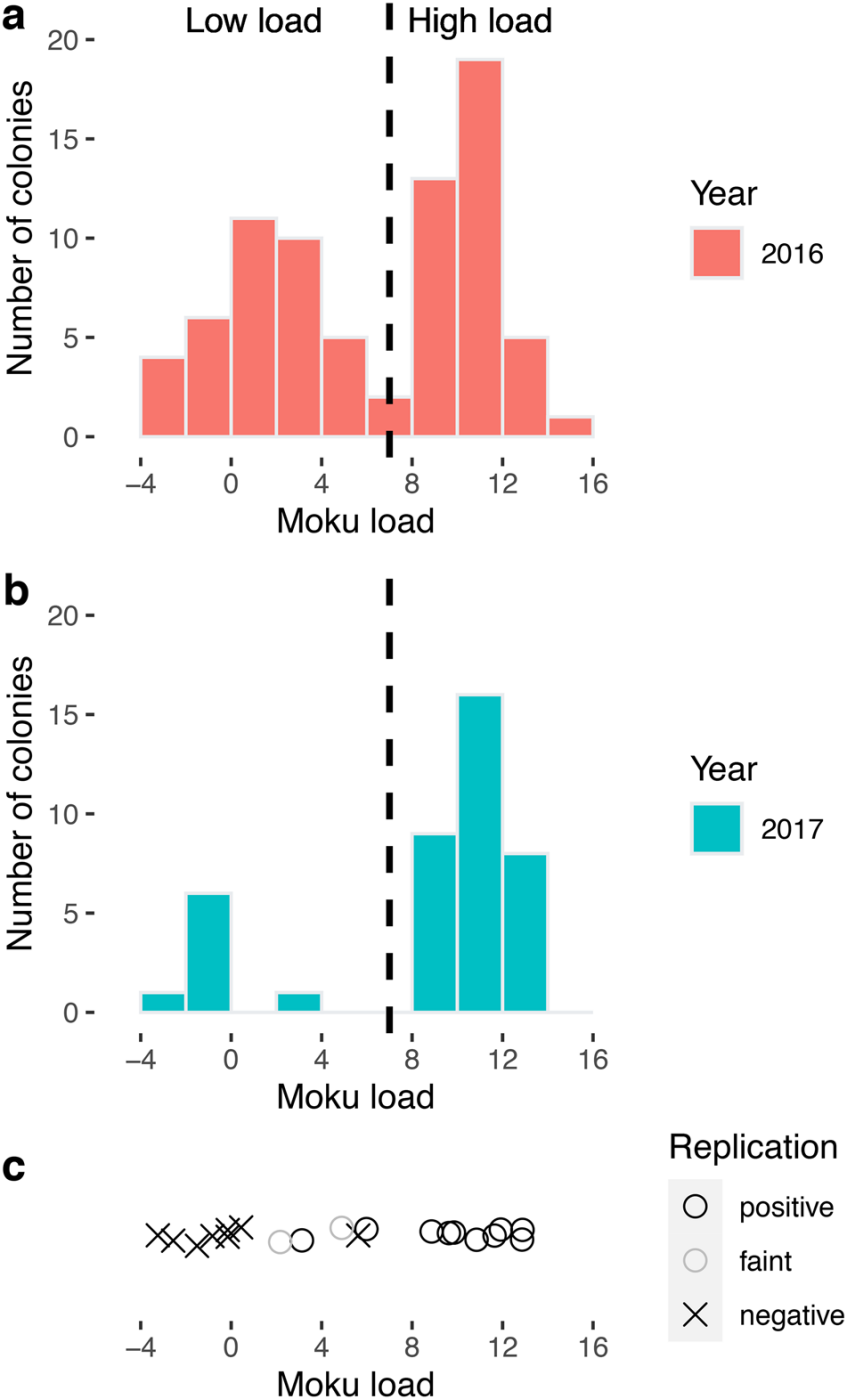
Moku virus load and replication in colonies of *Vespula pensylvanica* in 2016 **(a)** and 2017 **(b)**. Each sample is a pool of 20 workers from a single colony. Viral load is calculated from qPCR data as -(Cq_viral gene_ – Cq_control gene_)). Load is bimodal: high load colonies have roughly 1000-fold more copies of virus RNA than low load colonies (each load unit represents a twofold increase in viral RNA). Dashed line indicates the threshold (load = 7) used to separate “low” and “high” load colonies in analyses. c. Viral replication, determined by strand-specific reverse-transcription PCR for 20 representative colonies, was observed in a subset of samples with relatively high load. Open circles represent strong bands on an agarose gel, indicating a positive replication test. Grey circles represent very faint bands, which could indicate lower levels of replication. Crosses represent no band, and thus no detected replication. Points are jittered in the y dimension to improve visualization.

We detected *Arsenophonus* sp. and Moku Virus in all colonies assayed (2016: 76 colonies, and 2017: 37 colonies) across our study site in Hawaii Volcanoes National Park (Fig. 1), though the loads were quite variable. Pathogens were not analyzed in 2019 (Table 1). Trypanosomatids were detected only in 2016 in 6 of 76 colonies. We detected no associations between pathogens in either year (Pearson’s correlation coefficient < 0.1, p > 0.3 for each test; n = 76 colonies in 2016, n = 37 in 2017). Moku Virus loads were strongly bimodal in both years, and replication was detected mostly in colonies with high load (Fig. 2). To confirm target identity, we sequenced five representative PCR products from the positive *Arsenophenous* and trypanosomatid samples. All five *Arsenophonus* sp. sequences were identical (Genbank Accession # MW484946), and all five trypanosomatid sequences were also identical (Genbank Accession # MW925068).

### Honeybee hive density

Honeybee colonies were very abundant at the Hilina Pali site, located along Hilina Pali road south of Kilauea Crater (Fig. 1). In 2016, methodical searches of 28 partially overlapping 200 m radius circles (total 1.41 km^2^) centered on *V. pensylvanica* nests yielded 13 hives, and thus we observed a lower bound average density of ~ 9.2 hives/km^2^. In contrast, no honeybee hives were found at Kīpuka Kahali’i despite extensive searching (18 partially overlapping circles searched for a total of 0.61 km^2^). This absence is likely due to the lack of suitable nest cavities, as all trees were relatively young, the result of regrowth following the 1989 Mauna Ulu eruption, and all rock cavities filled with volcanic gravel from recent volcanic activity.

### Colony survival

**Figure 3:**
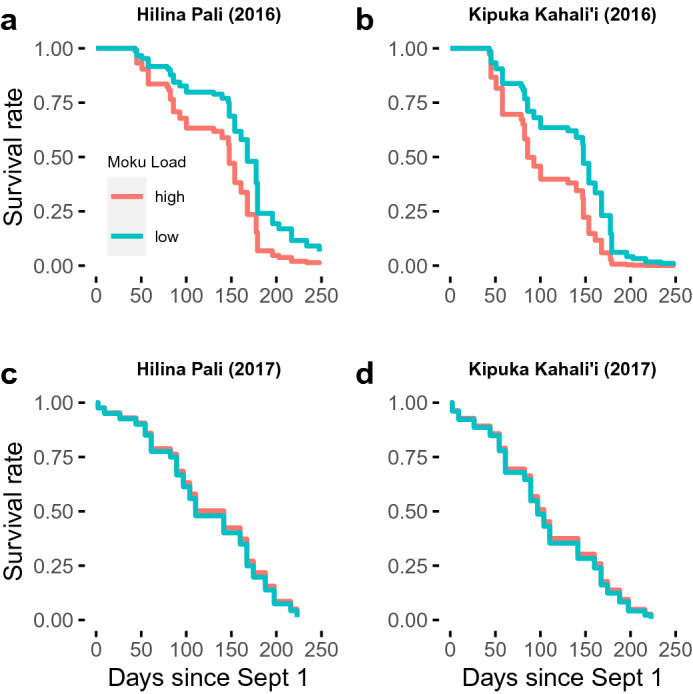
Adjusted survival curves showing the effect of Moku Virus load on survival for wild *Vespula pensylvanica* colonies in Hawaii Volcanoes National Park. In 2016, colonies with low Moku Virus load survived significantly longer than those with high load (Cox proportional hazards test with additional covariates; Table 1), with the Hilina Pali site **(a)** having significantly longer survival than the Kīpuka Kahali’i site **(b)**. Sample sizes in **(a)** are n = 23 and 27 for high and low load respectively, and in **(b)** are n = 15 and 9. The effect of Moku Virus was not observed in 2017 in either Hilina Pali **(c)** or Kīpuka Kahali’i **(d)**. Sample sizes are n = 25 and 5 for **(c)** and n = 5 and 1 for **(d)**, for high and low loads, respectively. These curves are adjusted to account for cox regression model covariates using the ggadjustedcurves() function in the *survminer* package and the models reported in Table 1.

Of 76 colonies that were monitored starting Oct 1, 2016, all but one senesced before May 5, 2017 (Fig. 3). The exceptional colony, HP-16-63, survived until February, 2018. Of the 41 colonies that were monitored starting Oct 1, 2017, all colonies senesced before April 8, 2018. Of the 40 monitored colonies in 2019, all but four died following extreme rainfall events in December, 2019 (Supplementary Fig. S1), and monitoring was discontinued in January as foraging levels were very low.

In a survival model of all 134 colonies included in the feeding experiment across the three years (2016, n = 53; 2017, n = 41; 2019, n = 40), feeding had no effect (*β* = 0.002, z = − 0.008, p = 0.99; Supplementary Fig. S2) on colony longevity relative to controls, while year had a significant effect, with 2019 colonies dying significantly earlier (*β *= 0.98, z = 3.88, p < 0.001). Additional predictors were not included in the global model, as they were not collected in all 3 years.

When analyzing survival for each year independently, we found that in 2016, colonies with low Moku Virus load (relative load < 7) survived significantly longer than those with high load (Fig. 3; Table 2). This effect was most conspicuous for the first 150 days of observation. We also observed a significant effect of site, with colonies at KK senescing earlier (Table 2). Finally, there was a *positive* effect of conspecific density in 2016, with colonies with more near neighbors surviving longer (Table 2). No effect of feeding or proximity to honeybee hives (Supplementary Table S1) was observed in 2016. In 2017, we observed no significant effects on survival, though the effect size for site was similar to that in 2016 and may have been significant if not for the relatively low sample size. Although feeding did not significantly extend colony survival in 2017 (Table 2; Figure S2), the effect was positive, and could perhaps have been significant with a larger sample size. There were no significant effects of experimental warming on longevity in either 2017 or 2019 (Table 2).

**Table 2:** Cox proportional hazard models of *V. pensylvanica* colony survival. *Indicates p < 0.05, **indicates p < 0.01; ***indicates p < 0.001.

Year	Colonies	Predictors	*β*	se(*β*)	*z*	*P*
2016	All (n = 74)	**Moku load (high)**	**0.73**	**0.25**	**2.85**	0.004**
		*Arsenophonus* sp. load	−0.06	0.03	−1.77	0.08
		Trypanosomatids (present)	−0.38	0.53	−0.73	0.47
		**Site (KK)**	**0.60**	**0.27**	**2.19**	0.03*
		**Wasp colony density**	−**0.21**	**0.10**	−**2.24**	0.02*
	Expt (n = 53)	Moku load (high)	0.56	0.31	1.78	0.08
		*Arsenophonus* sp. load	−0.02	0.04	−0.69	0.49
		Trypanosomatids (present)	−0.57	0.56	−1.03	0.30
		Treatment (feed)	0.43	0.29	1.49	0.14
		**Site (KK)**	**0.89**	**0.34**	**2.66**	0.007**
		Wasp colony density	−0.13	0.10	−1.27	0.20
2017	Expt (n = 37)	Moku load (high)	−0.07	0.52	−0.13	0.90
		*Arsenophonus* sp. load	0.04	0.03	1.28	0.20
		Feeding (fed)	−0.53	0.43	−1.24	0.22
		Site (KK)	0.53	0.46	1.15	0.26
		Wasp colony density	−0.11	0.29	−0.39	0.70
		Warming (coned)	0.21	0.38	0.57	0.57
2019	Expt (n = 41)	Feeding (fed)	0.08	0.34	0.23	0.82
		Warming (coned)	−0.14	0.34	−0.42	0.67

Note: bold lines indicate significant predictors. Positive coefficients (*β*) indicate a higher estimated hazard rate, i.e. reduced survival. Wasp colony density refers to the number of wasp colonies within 100 m of a focal colony. “Expt” colonies were those that were included in the feeding experiment in each year.

### Spatial patterns in pathogen load

**Figure 4:**
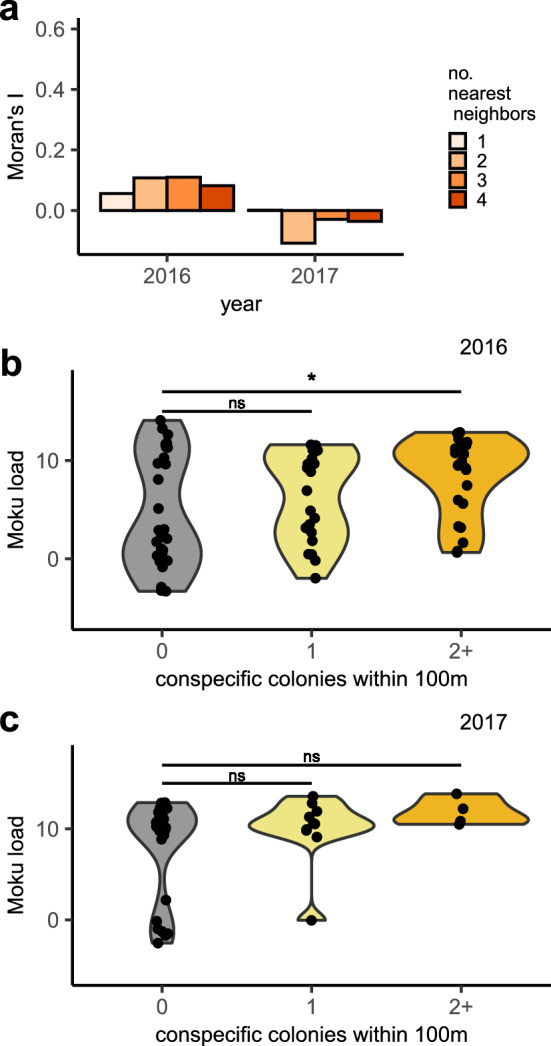
Spatial patterns in colony-level Moku virus load. **(a)** Moku load is not spatially autocorrelated, as Moran’s I was close to, and not significantly different from, zero, for nearest neighbors defined as the closest 1–4 colonies (p > 0.05 for all Moran’s I tests). **(b)** In 2016, colonies > 100 m from other conspecific colonies were significantly more likely to have low Moku loads than colonies with two or more close neighbors (binomial GLM; n = 74, z = 2.3, p = 0.02; “High” vs “Low” threshold relative load was 2; see Fig. 1, Supplementary Figure S1 and main text). **(c)** The same trend was observed in 2017, but the difference was not statistically significant. Violin plots, created using ggplot2, depict the density of points in each category. * indicates p < 0.05. “ns” indicates not significant.

Colony-level Moku Virus loads were not spatially autocorrelated (Fig. 4a; Supplementary Fig. S3), nor was viral load influenced by proximity to honeybee hives (68 colonies in 2016: GLM: High vs Low: t = -0.06, p = 0.95; High vs None: t = 0.57, p = 0.57). Arrival traffic rate, an index of colony size, did not predict Moku load at the time of sample collection (binomial GLM on 88 colonies in 2016 and 2017: z = 0.012, p = 0.99). We also observed no correlation between a colony’s 2017 load and the load of the previous year’s colony closest to the nest site (Pearson’s r = − 0.01, n = 38, p < 0.95). However, colonies with no conspecific neighbors within 100 m were more likely to have low Moku load in 2016, compared to colonies with more near neighbor nests (Fig. 4b; GLM: 0 vs 1 neighbor: z = 1.01, p < 0.31; 0 vs 2 + neighbors: z = 2.61, p < 0.009). The same trend was observed in 2017, but differences were not statistically significant (Fig. 4c; GLM: 0 vs 1 neighbor: z = 1.00, p < 0.31; 0 vs 2 + neighbors: z = 0.008, p < 0.99).

**Figure 5:**
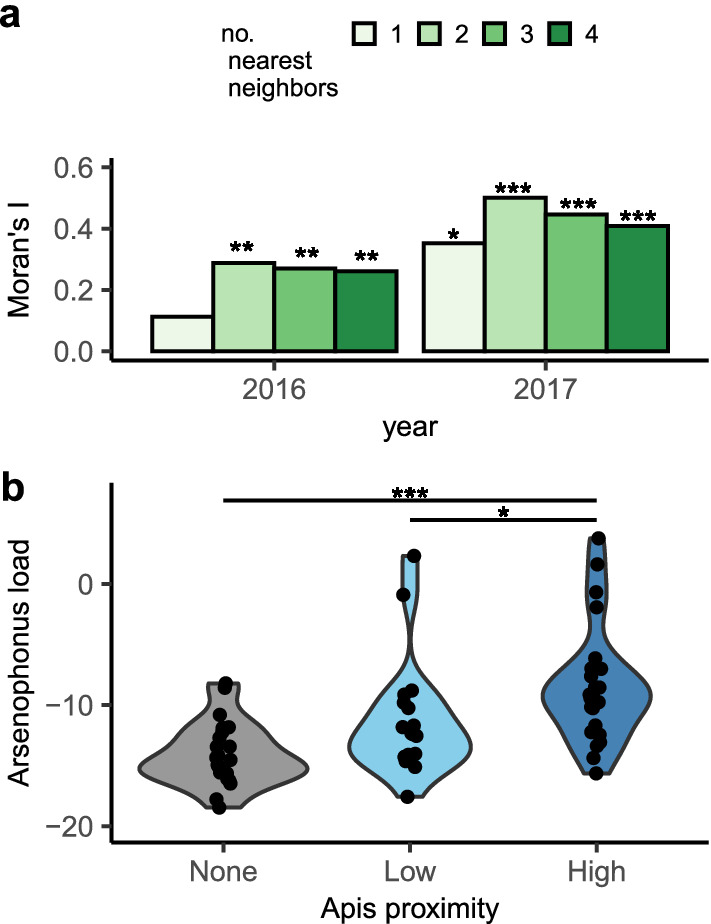
Spatial patterns in colony-level *Arsenophonus* sp. load. **(a)** *Arsenophonus* sp. loads are positively spatially autocorrelated, indicated by significantly positive Moran’s I tests for a variety of definitions of neighbors (k = 1–4 nearest neighbors). **(b)** Colony-level *Arsenophonus* sp. load is predicted by the proximity to feral honeybee hives in 2016. All “None” colonies were at the KK site, while “Low” and “High” honeybee colonies were at the HP site. Honeybee hives were not searched for in 2017. Violin plots produced in ggplot2. For map of *Arsenophonus* sp. load and honeybee hives, see Supplementary Figure S5. * indicates p < 0.05, ** indicates p < 0.01; *** indicates p < 0.001.

In contrast, *Arsenophonus* sp. bacterial loads were positively spatially correlated (Fig. 5a; Supplementary Fig. S4). The number of conspecific colonies within 100 m did not affect *Arsenophonus* sp. loads (2016 GLM: 0 vs 1 neighbor: t = − 0.58, p = 0.56; 0 vs 2 + neighbors: t = − 1.79, p < 0.08; 2017 GLM: 0 vs 1 neighbor: t = 1.82, p < 0.08; 0 vs 2 + neighbors: t = − 0.69, p < 0.45). However, proximity to honeybee hives was significantly associated with *Arsenophonus* sp. loads in 2016 (Fig. 5b; 68 colonies in 2016: GLM: High vs Low: t = − 2.27, p < 0.03; High vs None: t = − 4.81, p < 0.001).

We did not perform spatial statistics on trypanosomatid presence, given the low number of detections (only 6 positives in 2016, no positives in 2017). However, the locations of the positive colonies do suggest a positive spatial autocorrelation (Supplementary Fig. S5).

## Discussion

Here we examined how biotic, abiotic and spatial factors affect patterns in colony longevity. Our most important finding is the association between colony-level Moku Virus load and colony longevity in 2016. This effect was most pronounced in the first 100 days of colony monitoring (Fig. 3a,b), which makes sense given that load was estimated from a single collection at the start of colony monitoring; after 4 months, those initial loads are likely to have changed substantially. The correlative nature of our observation means that we cannot determine whether Moku Virus infections actively cause colony death, or instead if Moku Virus is merely more abundant in weakened colonies that will soon die as the result of other causes. However, it is clear that virus copy numbers in active infections are extremely high (KJL, unpublished data;^27^ ), and likely tax cellular resources as a result of such replication. Furthermore, we found no association between Moku load and colony size at the time of pathogen sampling (estimated from foraging traffic^38^). This suggests that Moku infections were not high only in weak colonies near death, rather load is associated with colony survival in both strong and weak colonies over the subsequent months.

What factors influence Moku Virus load? We know of no studies assessing transmission to date, though the virus has been detected in honeybees and their mites on Hawaii^27,39^, as well as in honeybees, *Vespa* hornets, and *Vespula* spp. wasps in Europe and New Zealand^40–43^. We found that although Moku is not significantly spatially autocorrelated, loads are significantly higher in colonies with a greater number of conspecific colonies within 100 m (Fig. 4). This suggests that transmission may occur primarily between conspecifics, rather than from other species, such as honeybees. Pollinator pathogens can be transmitted between adults on flowers^35^, via drifting into^34^ or raiding of^36,44^ conspecific colonies, and among adults and larvae via trophallaxis^45,46^. Notably, *V. pensylvanica* colonies in Hawaii are porous to non-nestmate queens and workers^8,47^, and drifting adults could transmit Moku Virus between colonies. Given that colony density and Moku Virus load have opposing effects on colony survival, there may be balancing selection on nest site selection by queens that trades off potential costs of higher pathogen load with whatever benefits accrue from nesting in areas with many conspecifics. Our findings are the first suggestion that this recently described virus may have important effects on colony survival and that nest densities in the field influence viral loads. These results motivate laboratory infections of multiple species to determine the degree to which this virus may affect important pollinator populations.

Our results for the bacteria *Arsenophonus* sp. contrast with those from the Moku Virus (Table 3). It appears that while Moku Virus is positively associated with *V. pensylvanica* density, suggesting intraspecific transmission, *Arsenophonus* sp. load is instead correlated with proximity to honeybee hives (Fig. 5), suggesting a role of interspecific transmission. Although *Arsenophonus* sp. is an intracellular endosymbiont, experiments in honeybees indicate that it is not transmitted to offspring via the egg, suggesting horizontal transmission among bees^48^. Honeybee hives are extremely abundant at the HP site (> 9 hives per square kilometer), much higher than most other estimates of *A. mellifera* densities reported in the literature, either directly observed (e.g., Refs^49,50^) or modeled from drone genetic diversity^51^. The positive spatial correlation of *Arsenophonus* sp. load may result from spillover from nearby honeybee hives, either via predation or sharing of floral resources. *V. pensylvanica* shares many pathogens with honeybees at this field site, and evidence from parallel changes in Deformed Wing Virus strains through time suggest active spillover from honeybees to *V. pensylvanica*^28^, though we know of no evidence to date of detrimental effects of these pathogens on wasps. *Arsenophonus* sp. has been negatively associated with honeybee hive health^31^, but whether infection has any consequences for *Vespula spp.* colonies in the wild remains to be determined.

**Table 3:** Summary of associations between pathogens and colony survival, density and spatial distribution.

	Associated with survival	Associated with wasp colony density	Spatially autocorrelated	Associated with honeybee proximity
Moku virus	Yes	Yes	No	No
*Arsenophonus* sp.	No	No	Yes	Yes
Trypanosomatids	No	No	Maybe	No

Most research on the effects of pollinator pathogens has focused on a handful of experimentally tractable species, often with controlled experimentation at the individual level in the laboratory^21,52–54^. Colony-level pathogen effects are often measured on artificially reared and maintained colonies^16,55^. Such experiments provide great insight by establishing causality and identifying the factors that modulate infections within colonies. However, it is unclear if and how these results translate to wild populations, and to species not amenable to laboratory rearing. Studies on wild in situ colonies^24^ complement laboratory experiments by identifying possible pathogen effects in wild populations, where pathogen dynamics may be quite different from those observed under laboratory conditions.

Counter to our expectations, neither experimental feeding nor nest warming significantly increased colony longevity during the course of our study. Based on observations from a decade earlier^15^, we had predicted that prolonged access to honeybee prey and honey would allow colonies to persist longer into the winter, and increase the chances that colonies would become perennial, persisting until the next summer. However, across all three years, we detected no effect of feeding on colony longevity. In 2016, neither direct feeding, nor colonies’ proximity to feral honeybee hives, increased longevity. In 2017, the positive but non-significant effect of feeding on longevity was consistent with preliminary data^15^, but in 2016 and 2019 the effects were weak and not in the predicted direction. Given the apparent variability among years, both within this study and in comparison to preliminary observations in 2006–08, it seems that if honeybees do serve as a diet supplement to *V. pensylvanica* that influences colony longevity, this only occurs under certain conditions not observed in this study.

Furthermore, it also appears that perenniality itself is likely variable between years and may be more rare than rates observed in previous decades. Of 76 colonies followed from 2016 to 2017, only one survived into the second season, and none of 41 tracked colonies discovered in 2017 persisted through the winter into the second season. Colony tracking was cut short in 2019 after a massive mortality event in December/January (Supplementary Fig. S1), likely the result of a period of exceptional precipitation, after which none of the four surviving colonies seemed likely to make it through the winter. In contrast, previous estimates of perenniality are variable but range up to 20% in this habitat^6,7,13,56^. It is important to note that we did observe colonies at our field sites that were omitted from experiments because they were likely perennial. They exhibited high traffic rates (> 200 forager arrivals per minute) and had multiple entrances with carton-lined tunnel mouths, all features of perennial colonies (E.W.R and K.J.L. unpub. obs). We observed 2, 2 and 1 such colonies in 2016, 2017 and 2019 respectively. Nearby, in Volcano Village, collections of colonies reported by the public suggest a higher rate of perennial colonies (Sankowitz et al., submitted), but large nests are more likely to be noticed and reported, and this bias also applies to the likelihood that they would be found in our nest searching as well. Thus, perennial colonies do occur at our site, and their low frequency in our tracked colonies relative to previous estimates based on encounters in the field may partially result from the methods of estimation. However, is also likely to be the result of variation in weather and other limiting factors, such as pathogens, prey and/or nectar availability, and volcanic activity.

Although true perenniality was rare in our study, we did observe substantial variation in annual colony longevity, and this longevity was significantly associated with site, colony density, and Moku Virus load in 2016. Given the differences in nesting substrate, honeybee presence, and forest composition between the Hilina Pali and Kīpuka Kahali’i sites, it is not surprising that wasp colony longevity differs between these two sites. However, this difference was only apparent in 2016, suggesting that the underlying causal factors vary between years, and highlighting the potential importance of fall and winter weather, also illustrated by the die-off associated with an extreme rainfall event in 2019. *Vespula pensylvanica* populations in Hawaii^7,56^ and on the mainland^57^ exhibit strong 2–3 year cycles in abundance that could reflect conditions also resulting in longevity differences between years. Interestingly, colony longevity is *positively* associated with conspecific colony density in 2016, measured as the number of *V. pensylvanica* colonies within 100 m, counter to a prediction based solely on inter-colony competition. This pattern could result from *V. pensylvanica* queens preferentially founding colonies in favorable areas, or differential mortality early in colony development that causes more failures in low quality areas, with these lower quality areas later limiting colony survival. Given that much of *V. pensylvanica* foraging occurs within a few hundred meters of the nest site^33^, landscape-level variation in resource availability is likely to create variation across nests in the availability of resources within foraging distance. Future experiments will be necessary to determine what factors create such a pattern and what factors yellowjacket foundresses use to assess habitat quality.

Our study provides support for a temporally variable effect of a putative pathogen, Moku Virus, on the longevity of *V. pensylvanica* colonies in the wild. However, the effect is small (a few weeks) relative to the large difference in longevity between annual and perennial colonies that originally motivated this study. Likewise, we saw variable effects of site and colony density, although this was inconsistent across years. A massive winter die-off following a major rain event in winter of 2019 demonstrated an important role of winter weather in explaining variation in colony survival. Future studies could compare longevity across sites and weather regimes to better understand variation in perenniality. It will also be important to study the role of social structure in colony longevity, given the facultative polygyny that occurs in populations in Hawaii (Hanna et al. 2014). Colonies with a single queen seem unlikely to survive into a second season, while colonies with multiple queens, including younger egg-layers, are more likely to survive through the winter. Understanding the emergent perennial life history will require investigating the complex interactions of a host of biotic (e.g. disease, density, social structure) and abiotic (e.g. rainfall, temperature) factors, which together likely influence colony longevity in this important invasive species.

## Supplementary Information

Supplementary Information.

## Data Availability

Data from this study is available on Dryad (https://doi.org/10.6086/D12Q32).
