# Author Correction: Viral load, not food availability or temperature, predicts colony longevity in an invasive eusocial wasp with plastic life history

**DOI:** 10.1038/s41598-021-96295-7

**Published:** 2021-08-16

**Authors:** Kevin J. Loope, Erin E. Wilson Rankin

**Affiliations:** 1 Department of Entomology, University of California, Riverside, 900 University Ave., Riverside, CA 92507 USA; 2 Present Address: Department of Biology, Georgia Southern University, 4324 Old Register Road, Statesboro, GA 30460 USA

Correction to: *Scientific Reports* 10.1038/s41598-021-89607-4, published online 12 May 2021

The Acknowledgments section in the original version of this Article was incomplete.

“We thank Patrick Fuller, Stephanie Gayle, Twylah Morelli, Jessica Purcell, Madison Sankowitz, Mari West, and especially David Rankin for assistance during fieldwork. We also thank Jeff Judd for providing honeybees. We are grateful to Hoang Vuong, Jason Rothman and Quinn McFrederick for advice regarding lab methods, and to Julie Lee and Giselle Lozano for assistance in the lab. Fieldwork was conducted under permits from the National Park Service: HAVO-2016-SCI-0050 and HAVO-2019-SCI-0021. This work was supported by the US NSF (DEB #1557163) to EWR and a USDA NIFA postdoctoral fellowship (2016-67012-24681) to KJL.”

now reads:

“We thank Patrick Fuller, Stephanie Gayle, Twylah Morelli, Jessica Purcell, Madison Sankowitz, Mari West, and especially David Rankin for assistance during fieldwork. We also thank Jeff Judd for providing honeybees. We are grateful to Hoang Vuong, Jason Rothman and Quinn McFrederick for advice regarding lab methods, and to Julie Lee and Giselle Lozano for assistance in the lab. Fieldwork was conducted under permits from the National Park Service: HAVO-2016-SCI-0050 and HAVO-2019-SCI-0021. This work was supported by the US NSF (DEB #1557163) to EWR and a USDA NIFA postdoctoral fellowship (2016-67012-24681) to KJL. The authors also acknowledge funding from the US NSF (DEB # 1655963) to EWR.”

The original Article has been corrected.

