# Supplementary material for: Viral load, not food availability or temperature, predicts colony longevity in an invasive eusocial wasp with plastic life history
Source: Sci Rep. 2021 May 12;11:10087. doi: 10.1038/s41598-021-89607-4 (PMC8115236; doi:10.1038/s41598-021-89607-4)
Supplement: Supplementary file 1 — Supplementary Information. [file 41598_2021_89607_MOESM1_ESM.docx]

**SUPPLEMENTARY MATERIALS for**

**Viral load, not food availability or temperature, predicts colony longevity in an invasive eusocial wasp with plastic life history**

**Kevin J. Loope^1,2*^ and Erin E. Wilson Rankin^1*^**

^1^Department of Entomology, University of California, Riverside, 900 University Ave. Riverside, CA 92507

^2^ current address: Department of Biology, Georgia Southern University, 4324 Old Register Road

Statesboro, GA 30460

*****corresponding authors. KJL: kjloope@gmail.com, EEWR: erin.rankin@ucr.edu

**Table of Contents**

| Section | Page |
| --- | --- |
| Supplementary Tables | 2 |
| Supplementary Table S1: Cox survival model including honeybee proximity | 3 |
| Supplementary Table S2: Forager traffic and sample size for treatment groups in feeding experiment. | 3 |
| Supplementary Table S3: Primers used in this study | 3 |
|  |  |
| Supplementary Figures | 4 |
| Supplementary Figure S1: Survival curves for each year. | 4 |
| Supplementary Figure S2: Effect of experimental feeding on Vespula colony survival. | 4 |
| Supplementary Figure S3: Map showing Moku Virus loads | 5 |
| Supplementary Figure S4: Map showing *Arsenophonus* sp. loads and honeybee colonies | 6 |
| Supplementary Figure S6: Map showing trypanosomatid detection | 7 |
| Supplementary Figure S7: Field site and experimental methods. | 8 |
| Supplementary Figure S8: The presence of passive heating fiberglass cones increased mean nest entrance temperatures. | 9 |
|  |  |
| Supplementary Methods | 10 |
| References for Supplementary Materials | 11 |

**Supplementary Table S1:** Cox survival model of survival in 2016 including honeybee proximity (n = 68 colonies). Bold lines indicate significant predictors.

| **predictors** | ***β*** | **se(*β*)** | ***z*** | ***p*** |
| --- | --- | --- | --- | --- |
| **Moku load (high)** | **0.81** | **0.27** | **2.99** | **0.003** |
| *Arsenophonus* sp. load | -0.04 | 0.03 | -1.11 | 0.26 |
| Trypanosomatids | -0.49 | 0.54 | -0.91 | 0.36 |
| honeybees (none) | -0.37 | 0.35 | -1.05 | 0.29 |
| honeybees (low) | 0.59 | 0.33 | 1.78 | 0.07 |
| Wasp colony density | -0.13 | 0.10 | -1.28 | 0.20 |

**Supplementary Table S2**. Forager traffic and sample size for treatment groups in feeding experiment. Forager traffic (mean number of arrivals per minute) is an index of colony size. The number of colonies in each group is in parentheses.

|  |  | 2016 |  | 2017 | 2019 |
| --- | --- | --- | --- | --- | --- |
| *Apis* proximity | None (KK) | Low (HP) | High (HP) | Not assessed | Not assessed |
| Fed | 26.7 (9) | 25.8 (8) | 22.1 (9) | 22.1 (20) | 22.4 (21) |
| Control | 25.0 (9) | 22.0 (9) | 24.4 (9) | 22.1 (20) | 22.1 (20) |

**Supplementary Table S3:** Primers used in this study

| **Target** | | **Use** | **Primer** | | | **Sequence** | | | | **Source** | | |  |
| --- | --- | --- | --- | --- | --- | --- | --- | --- | --- | --- | --- | --- | --- |
| Moku Virus | qPCR | | | | MVF | | | GACTGTTTAAAGGATTACCG | | | Ref ^1^ | | |
|  |  |  |  |  | MVR | | | GCACCTCTATAAGCAGAGAG | | |  |  |  |
| *Arsenophonus* sp. | qPCR | | | | yaeT-qF | | | TCGAGCGCTATTTTCAACG | | | Ref ^2^ | | |
|  |  |  |  |  | yaeT-qR | | | GTTGGCCGCTCTTTTACTTG | | |  |  |  |
| *Arsenophonus* sp. | PCR | | | | YaeTf | | | GCATACGGTTCAGACGGGTTTG | | | Ref ^3^ | | |
|  |  |  |  |  | YaeTr | | | GCCGAAACGCCTTCAGAAAAG | | |  |  |  |
| Trypanosomatids | PCR | | | | CB-SSU rRNA-F2 | | | CTTTTGACGAACAACTGCCCTATC | | | Ref ^4^ | | |
|  |  |  |  |  | CB-SSU rRNA-B4 | | | AACCGAACGCACTAAACCCC | | |  |  |  |
| *eIF3* | qPCR | | | | eIF3-S8-fwd | | | AGAAGAGTATGGCTGATGGTGA | | | Ref ^5^ | | |
|  |  |  |  |  | eIF3-S8-rev | | | TCACTCTCTGAATCGCTACCC | | |  |  |  |
| Moku Virus negative strand RNA | Strand specific RT-PCR | | | | tagMVF | | | agcctgcgcaccgtggGACTGTTTAAAGGATTACCG | | | Ref ^1,6^ | | |
|  |  | | |  | | |  | |  | | |  |  |

**Supplementary Figures**

**Supplementary Figure S1:** Survival curves for each year. The triangles indicate the timing of large precipitation events (green: 2017, blue: 2019). In each event, over 50 cm of rain fell in 4 days at a rain gauge station in Volcano, HI, approximately 10 km from our field sites.

**Supplementary Figure S2:** Effect of experimental feeding on Vespula colony survival.


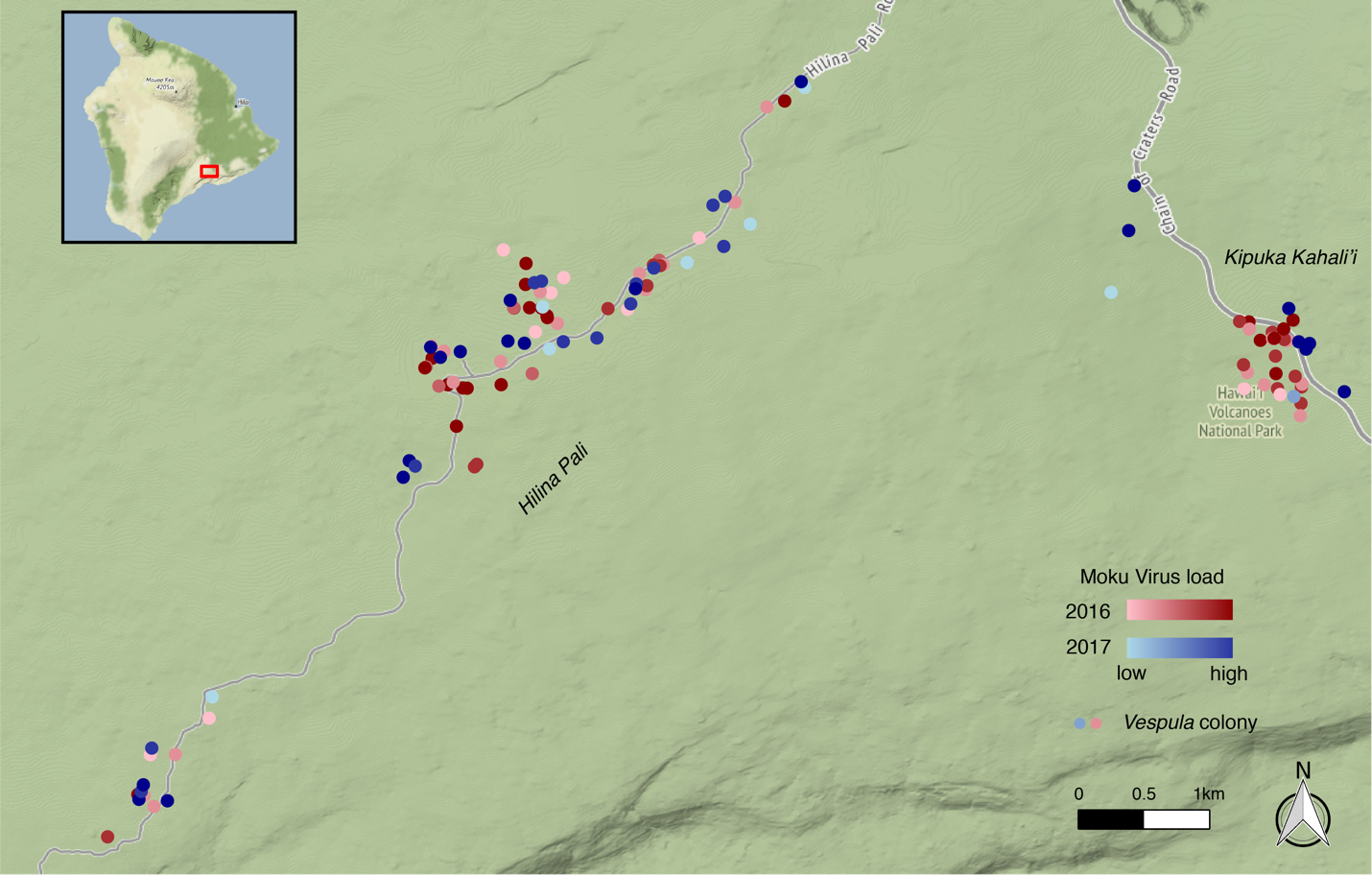


**Supplementary Figure S3:** Map showing Moku Virus loads along Hilina Pali Road, and at Kīpuka Kahali’i (along Chain of Craters Road) in Hawaii Volcanoes National Park on the Big Island of Hawaii. Not shown are two colonies from Crater Rim Trail (2016) which were omitted from spatial analyses. Basemap: Stamen Terrain (obtained through package *ggmap*).

**
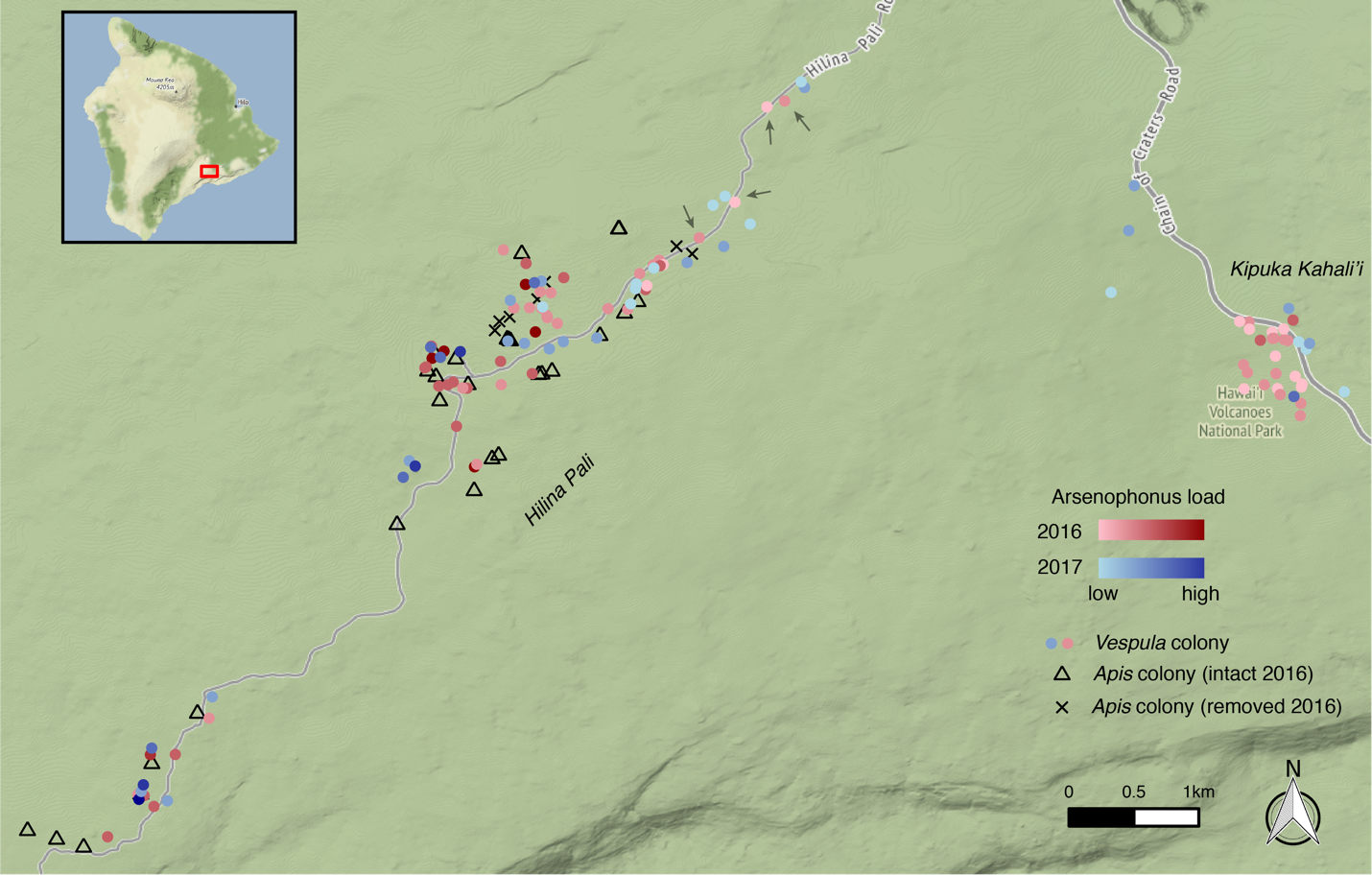
**

**Supplementary Figure S4:** Map showing *Arsenophonus* sp. loads and honeybee colonies along Hilina Pali Road, and at Kīpuka Kahali’i (along Chain of Craters Road) in Hawaii Volcanoes National Park on the Big Island of Hawaii. Not shown are two colonies from Crater Rim Trail (2016) which were omitted from spatial analyses. Arrows indicate four colonies from 2016 for which honeybee proximity was unknown; these were omitted from honeybee proximity analyses. Basemap: Stamen Terrain (obtained through package *ggmap*).

**
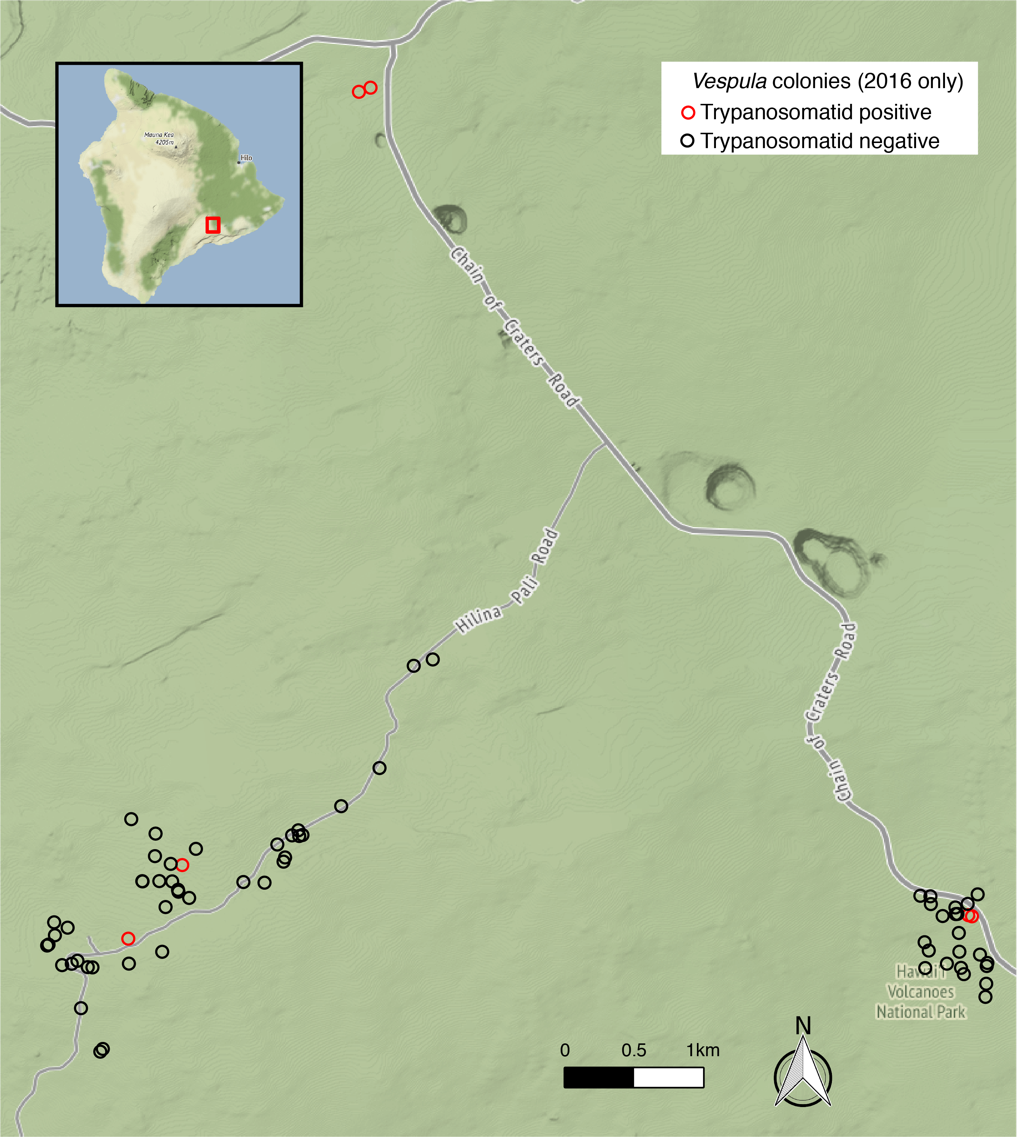
**

**Supplementary Figure S6:** Map showing trypanosomatid detection in *V. pensylvanica* colonies along Hilina Pali Road, at Kīpuka Khali’I and at Crater Rim Trail in Hawaii Volcanoes National Park. All colonies depicted were sampled in 2016, as no positives were detected in 2017 colonies. Basemap: Stamen Terrain (obtained through package *ggmap*).


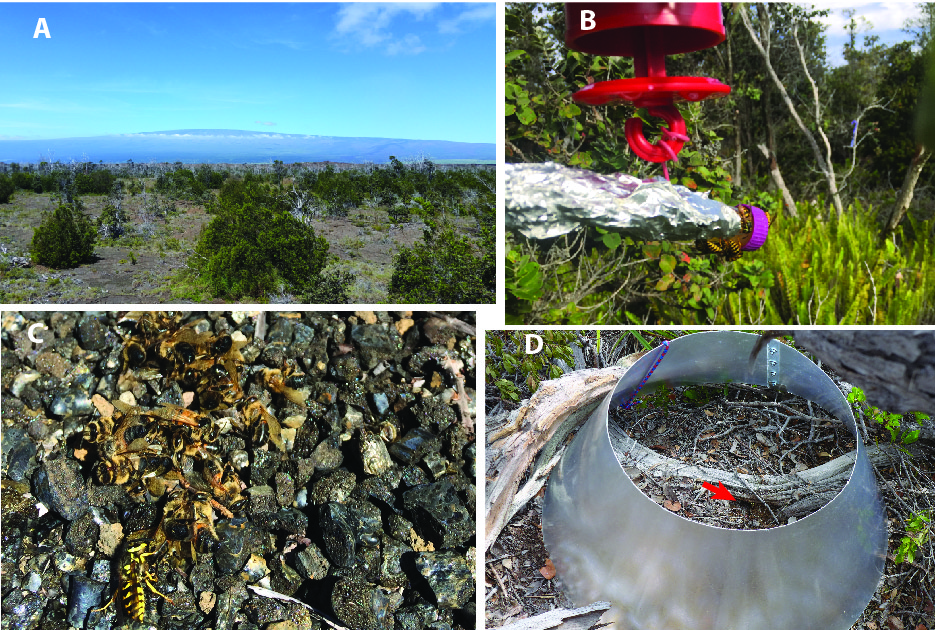


**Supplementary Figure S7:** Field site and experimental methods. a. Typical habitat along Hilina Pali Road, in Hawaii Volcanoes National Park. b. A 15 ml honey feeder with foraging wasps. The feeder is suspended from an ant guard (Perky-Pet) attached to a low branch within 10m of the nest entrance. c. A foraging wasp processing a dead honeybee in a feeding trial. d. A passive heating cone in place. The nest entrance tunnel is indicated by the red arrow at center. All photos by KJL.

**Supplementary Figure S8:** The presence of passive heating fiberglass cones increased mean nest entrance temperatures in 2017. Temperature data were collected using iButtons placed ~5cm inside of the nest entrance hole to avoid exposure to direct sunlight. iButtons collected temperature data every 3 hours from Oct 1 until the colony died. Sample sizes (nests) reported within each bar. Error bars are standard errors. * indicates p<0.05, ** indicates p < 0.001 in two-tailed t-tests.

**Supplementary Methods**

**Experimental feeding**

Honeybee adults were collected from hives using a vacuum, killed by freezing and frozen until use. Bees were thawed, coated in ~2 ml juice from cans of chicken (Hormel Foods) to make them immediately attractive for *V. pensylvanica* foragers, and placed ~10cm from the nest entrance. Preliminary trials showed that protein-foraging colonies would find and drag such bees into the nest within a few hours of their placement. Honey was provided in a 15 ml feeder placed within 5 m of the colony entrance, wrapped in tin foil and refilled weekly as needed. Feeding continued until colonies died.

*V. pensylvanica* colonies were assigned to treatments based on colony size, estimated using the number of foragers arriving in 2-minute counts on 1-2 fair-weather days in late September (Supplementary Table S2; Malham et al. 1991). In 2016, we balanced assignments across three *Apis* densities: no *Apis* (KK site), low *Apis* (HP site but >200m from a known *Apis* hive), and High Apis (HP site and <200m from an Apis hive). In 2017 and 2019, we did not assess proximity to the nearest *Apis* hive, and instead assigned colonies to treatment by ordering them by traffic rate and then alternating assignments to treatment and control. In all years we excluded obviously perennial colonies with >150 arrivals per minute.

**Passive heating cone design**

Perspex cones were created using the cone design of Marion et al. ^8^. Cones had an opening of 50 cm, a sloped angle of 60°, and a bottom diameter of 84.6 cm. Metal bolts with washers and nuts were used to hold the cone in shape (Fig. S7).

**Pathogen detection and quantification**

For 2016 samples, RNA was extracted by homogenizing a pool of 20 adult workers per colony using a rolling pin and plastic bag, followed by phenol/chloroform extraction using the protocol described by Evans et al. (2013). For 2017 samples, a pool of 20 workers was homogenized while flash frozen in liquid N2 using a Geno/Grinder (SPEX), and then aliquots of this frozen homogenate were extracted using TriSURE (Bioline) following standard protocols. DNA was extracted from homogenate using a Qiagen DNeasy Kit. Extracts were analyzed with a Nanodrop for purity, and those with 260:280 or 230:280 ratios less than 1.6 were re-extracted. No pathogen data was collected for 2019 colonies.

Quantitative PCR was performed using a Biorad CFX Real Time PCR machine. All samples were run in duplicate and Cq values averaged. To quantify Moku Virus , we used Luna OneStep RT-qPCR kits (New England Biolabs) according to manufacturer instructions, with 10ul reaction volumes and 10ng of RNA per reaction (for primers, see Supplementary Table S3; Ref ^1^). To quantify *Arsenophonus* sp. sp., we used a SSOAdvance SybrGreen Universal kit (Biorad), 10ul reaction volumes and 10ng DNA per reaction (for primers, see Supplementary Table S2; Ref ^2^). For each DNA and RNA sample we also quantified the reference gene *eIF3* (for primers, see Supplementary Table S3; Ref ^5^). Amplification efficiencies for all targets were between 90-110% for a 10-fold dilution series spanning the range of observed sample Cq values. For a quantitative index of pathogen loads, we used –ΔCq values (i.e., – (Cq­_pathogen gene_ – Cq­_control gene_)). Thus the pathogen load measures are on a log scale, with higher load represented by a higher score, and an increase of 1 corresponding to a doubling of pathogen DNA or RNA. For five representative samples, we amplified and Sanger sequenced a 471 bp region of the *Arsenophonus* sp. gene *YaeT* (Supplementary Table S3; Ref ^3^) to confirm target identity. To screen for trypanosomatids, we performed 10ul PCR reactions using GoTaq Green (Promega) with 20ng of extracted DNA (Primers: CB-SSU rRNA-F2, CB-SSU rRNA-B4; Supplementary Table S3, Ref ^4^). We then determined presence/absence of trypanosomatids by running PCR products on 1.4% agarose gels. Reference gene qPCR products from the same DNA samples served as positive controls for DNA quality. Five trypanosomatid PCR products were Sanger sequenced to confirm target identity.

**Moku Virus replication**

We performed strand specific RT-PCR to determine whether Moku Virus was replicating in our samples. Moku is a positive strand virus and thus the negative strand is only present during viral replication ^6^. We selected a subset of samples from across the distribution of viral loads by picking two samples from each bin of 2 cq values from our observed range of 8 to 28cq, for a total of 20 samples (Figure 2). We performed reverse transcription using 5ul reactions of ProtoScript II (New England Biolabs) following manufacturer’s instructions, with 150ng of RNA, 0.25ul of 10uM 5’ tagged MVF primer (﻿tag: agcctgcgcaccgtgg; Ref ^6^) and a 60 min incubation at 48 C. After reverse transcription, the primer was removed using a Cyclepure Cleanup Kit (Omega Bio-tek). We then performed PCR using the tag and the MVR primer, and ran PCR products on 2% agarose gels. We checked for false positives by running no-template controls, as well as no-RT, and no-RT-primer controls for each positive sample ^6^. None of these controls amplified.

**REFERENCES**

1. Highfield, A. *et al.* Detection and Replication of Moku Virus in Honey Bees and Social Wasps. *Viruses* **12**, 607 (2020).

2. Yañez, O., Gauthier, L., Chantawannakul, P. & Neumann, P. Endosymbiotic bacteria in honey bees: *Arsenophonus* spp. are not transmitted transovarially. *FEMS Microbiol. Lett.* **363**, fnw147 (2016).

3. Jousselin, E., d’Acier, A. C., Vanlerberghe-Masutti, F. & Duron, O. Evolution and diversity of *Arsenophonus* endosymbionts in aphids. *Mol. Ecol.* **22**, 260–270 (2012).

4. Schmid-Hempel, R. & Tognazzo, M. Molecular Divergence Defines Two Distinct Lineages of *Crithidia bombi* (Trypanosomatidae), Parasites of Bumblebees. *J. Eukaryot. Microbiol.* **57**, 337–345 (2010).

5. Dobelmann, J. *et al.* Fitness in invasive social wasps: the role of variation in viral load, immune response and paternity in predicting nest size and reproductive output. *Oikos* **126**, 1208–1218 (2017).

6. de Miranda, J. R. *et al.* Standard methods for virus research in *Apis mellifera*. *J. Apic. Res.* **52**, 1–56 (2013).

7. Malham, J. P., Rees, J. S., Alspach, P. A., Beggs, J. R. & Moller, H. Traffic rate as an index of colony size in *Vespula* wasps. *New Zeal. J. Zool.* **18**, 105–109 (1991).

8. Marion, G. M. *et al.* Open-top designs for manipulating field temperature in high-latitude ecosystems. *Glob. Chang. Biol.* **3**, 20–32 (1997).
